# Variations in bacterial profiles associated with semen collection timing and bull breed, analyzed using 16S rRNA sequencing and MALDI-TOF MS

**DOI:** 10.3389/fvets.2025.1583136

**Published:** 2025-09-05

**Authors:** Aleksandar Cojkic, Adnan Niazi, Ingrid Hansson, Jane M. Morrell

**Affiliations:** ^1^Department of Clinical Sciences, Swedish University of Agricultural Sciences (SLU), Uppsala, Sweden; ^2^Department of Animal Biosciences, Swedish University of Agricultural Sciences, Uppsala, Sweden; ^3^SLU Global Bioinformatics Centre, Swedish University of Agricultural Sciences, Uppsala, Sweden

**Keywords:** bull semen, bacteria, microbiome, breed, 16s sequencing, MALDI TOF-MS

## Abstract

**Introduction:**

Bacterial contamination can occur at multiple stages of semen processing, necessitating the use of antibiotics in bull semen preservation, mandated by regulatory guidelines. To manage antimicrobial resistance (AMR), targeted antibiotic use based on bacterial identification is essential. This study aimed to characterize bacterial communities in bull semen using metagenomic analysis and MALDI-TOF MS across different semen collection times from the same bulls and between two breeds.

**Methods:**

Semen samples were collected from 20 dairy bulls (8 Viking Holstein and 12 Viking Red). Each bull provided three ejaculates within a week: the first after a 96 h since previous collection (T1), the second 48 h later (T2), and the third 24 h after the second (T3). Bacterial species were identified through culturing on cattle blood agar, followed by MALDI-TOF MS identification. Additionally, 16S rRNA sequencing was performed to determine bacterial diversity after DNA extraction.

**Results:**

MALDI-TOF analysis identified 33 bacterial species across 60 semen samples. Six species were exclusive to Viking Holstein (VH) bulls, while 12 were specific to Viking Red (VR) bulls. Certain bacterial species were present only at specific time points: three in the first ejaculate, seven in the second, and five in the third. Across individual bulls, *Bacillus* spp., *Proteus* spp., and *Staphylococcus* spp. were the most consistently detected. Metagenomic analysis revealed 23 phyla and 402 genera in semen samples. Alpha diversity (Shannon index) showed a trend toward *p* = 0.07 across the bull samples, while beta diversity significantly differed between breeds, with VH samples forming a distinct cluster and VR samples displaying greater microbiome variability. Additionally, specific genera appeared only at one collection time point: *Bacteroides, Serratia, Pantoea* at T1, *Wolbachia, Prevotella, Peptococcus, Alloprevotella* at T2, and *Streptococcus, Staphylococcus*, and *Mycoplasma* at T3. Specific genera, *Acidocella* and *Escherichia*, exhibited negative correlations with most bacterial taxa but showed a slight positive correlation with each other; while *Acidocella* was detected in nearly all semen samples, except for two samples.

**Discussion:**

The seminal microbiota of bulls varies over time and differs between breeds, indicating that it is influenced by a complex interaction of environmental, physiological, and host-related factors.

## 1 Introduction

Semen can never be completely free of bacteria (1). Bacterial contamination can occur at various stages of semen processing, influenced by factors such as bull preputial hygiene, processing of semen samples, packaging, and storage (1). Bacteria present in semen samples can have both direct and indirect effects on sperm quality, affecting sperm motility, viability, and fertilization potential. Understanding not only the presence of bacteria but also the bacterial load in semen samples is crucial, as it can influence sperm quality and depends on several factors. One significant factor is semen collection. To minimize the bacterial contamination during sampling, all equipment should be sterile, and bulls are typically teased before collection to stimulate the production of pre-seminal liquid, which helps clear the urethra (2). To mitigate the adverse effects of bacteria, legal regulations often mandate the addition of antibiotic combinations during bull semen preservation to inhibit bacterial growth and spreading of pathogenic bacteria (3). However, extensive use of antibiotics can contribute to the development of antimicrobial resistance (AMR).

To effectively manage and control AMR, it is crucial to use appropriate antibiotics targeting specific bacterial species (4). This necessitates the accurate identification of the bacteria present in bull semen samples. Various methods have been developed for bacterial identification. Initially, the isolation and identification of bacterial species in bull semen relied on culture-based methods and characterization through morphological and biochemical properties (5). As science progressed, additional identification techniques were introduced, enhancing the accuracy and efficiency of bacterial detection. In recent decades, metagenomic methods, such as 16S sequencing, have gained prominence as they provide detailed information about the bacterial DNA present in samples. Recent research has highlighted the relevance of the bovine microbiome in reproductive performance. Webb et al. (6) demonstrated dynamic and distinct microbial profiles in bull semen compared to feces over time. Gupta et al. (7) reviewed microbial shifts in the female reproductive tract linked to fertility outcomes. Additionally, Kilama et al. (8) showed that integrating seminal microbiota with genomic data greatly improved prediction of sperm quality traits. These studies support the biological importance of the seminal microbiome and underscore the need for further investigation under controlled conditions. Although these methods offer precise results regarding bacterial presence, they do not provide information about bacterial viability (9). To address this, Matrix-Assisted Laser Desorption/Ionization Time-of-Flight Mass Spectrometry (MALDI-TOF MS) can be employed, allowing bacterial identification up to the species level after culturing (10). To enhance taxonomic resolution and biological relevance of 16S rRNA gene sequencing with MALDI-TOF MS, which enables species-level identification of cultivable bacteria, providing a culture-based validation of sequencing results. While 16S sequencing captures broad microbial diversity, including non-culturable taxa, MALDI-TOF enables species-level identification of viable isolates, offering complementary strengths for characterizing the seminal microbiota. Previous findings suggest that factors such as duration of abstinence and individual bull characteristics influenced the total bacterial count of ejaculates (11). However, the role of abstinence time and differences in bacterial communities in this process are not yet well understood.

The primary aim of this study was to evaluate differences in the occurrence of bacterial species in ejaculates collected at different times from the same bulls using both 16S rRNA sequencing and MALDI-TOF MS for bacterial identification. An additional aim was to investigate differences between breeds.

## 2 Materials and methods

### 2.1 Semen samples

Semen samples were obtained from 20 dairy bulls (8 Viking Holstein and 12 Viking Red breeds), aged between 1 and 4 years, housed at the VikingGenetics Artificial Insemination Station in Skara, Sweden. Semen from each bull was collected three times within a single week. The initial ejaculates were collected at the start of the week, following a 96 h interval from the previous collection. The second collection occurred 48 h after the first, and the third was conducted 24 h following the second. After collection, each sterile collection tube containing the ejaculate was transferred immediately to the laboratory. Semen samples were then diluted at a 1:1 ratio with Andromed semen extender without antibiotics (AndroMed® CSS one-step, 200 mL; Minitüb GmbH, Tiefenbach, Germany). Approximately 5–10 mL of this diluted semen was transported overnight to the Swedish University of Agricultural Sciences laboratory, maintained at 6°C in an insulated box with a cold pack.

### 2.2 Bacteriology

#### 2.2.1 Bacterial analyses

For bacterial analyses, 1 mL of sperm sample was mixed with the same amount of peptone diluent (1 g peptone and 8.5 g NaCl per liter Milli-Q H_2_O, autoclaved at 121°C for 15 min). Subsequently, 0.1 mL of this mix was surface plated at cattle blood agar plates (Swedish Veterinary Agency- SVA, Uppsala, Sweden) which were then incubated at 37 ± 1°C with 5% CO_2_ and examined for bacterial growth after 24 h. Further examination of the plates was not possible due to the overgrowth of certain bacterial species, which compromised the identification of additional isolates. Bacterial colonies of different macromorphologies were recultured on new cattle blood agar plates and incubated for 24 h at 37 ± 1°C with 5% CO_2_ to obtain a pure culture. The colonies from the pure culture were then identified at the species level by matrix-assisted laser desorption ionization–time of flight mass spectrometry (MALDI-TOF MS) (Bruker Daltonics, Billerica, MA, USA). Score values between 2.0 and 3.0 were considered accurate at both genus and species levels, whereas score values between 1.7 and 2.0 were considered reliable only at the genus level. A score value between 0 and 1.7 means that identification was not possible since the peak of the unknown isolate does not represent any bacterium in the reference library.

#### 2.2.2 DNA extraction

The AllPrep DNA/RNA/miRNA Universal Kit (Cat No./ID 80224) was applied in accordance with the manufacturers protocol for the simultaneous purification of genomic DNA and total RNA, specifically to extract DNA from semen samples at the Swedish University of Agricultural Sciences (SLU). For each sample, a volume of 50 μL of sperm sample was centrifuged, and the supernatant was discarded, retaining only the pelleted cells. The DNA concentration and purity were subsequently assessed using the Qubit 1X dsDNA HS Assay Kit (Thermo Fisher Scientific, Eugene, Oregon, USA), within a quantitation range from 0.1 to 120 ng. DNA samples were then stored at −80°C until they were prepared for further analyses. Prior to submission for 16S rRNA amplification and sequencing, each sample was adjusted to a concentration of 0.4 ng/μL of DNA using an elution buffer. To mitigate the risk of contamination, a negative PCR control was conducted for the water used in DNA extraction and amplification. Additionally, the negative control for the DNA extraction kit was assessed using Qubit prior to sequencing, confirming the absence of detectable DNA.

#### 2.2.3 16S rRNA amplification and sequencing

The library preparation for the V3-V4 regions of 16S rRNA involved two sequential PCR amplifications, each followed by bead-based purification. These steps were conducted using the Agilent NGS Workstation Bravo (Agilent Technologies, USA) in a 96-well plate format to ensure precision and consistency. The first PCR (PCR1) was performed to amplify the 16S rRNA region from the bacterial DNA. Each 25 μL PCR1 reaction contained 4 ng of sample DNA, 12.5 μL KAPA HiFi HotStart ReadyMix (Cat no: 07958935001, Roche), 0.5 μg/μL bovine serum albumin (BSA) (Cat no: B14, Thermo Scientific; 50 mg/mL), 1.25 μL of a 7.5 μM primer mix (containing forward primer 341F and reverse primer 805R), and 0.5 μL dimethyl sulfoxide (DMSO). Following PCR1, a bead-based purification step was carried out using MagSi-NGS Prep Plus (Cat no: MDKT00010075, Tataa) to remove free primers and prepare the amplicons for the second PCR. This purification involved binding the DNA to magnetic beads, washing, and eluting it in elution buffer (EB) (Cat no: 19086, QIAGEN). The second PCR (PCR2) was performed to incorporate sample-specific indices for sequencing. Each 20 μL PCR2 reaction contained 6 μL of purified amplicon, 10 μL KAPA HiFi HotStart ReadyMix (Cat no: 07958935001, Roche), and 4 μL of an indexing primer mix (i5 and i7 indexing primers, 2.5 μM). The PCR conditions and primers used for V3-V4 amplification are detailed in Supplementary Table 1. A final bead-based purification step using MagSi-NGS Prep Plus (Cat no: MDKT00010075, Tataa) was performed to remove any remaining free primers and further purify the amplicons. The quality of the adapter-ligated libraries was assessed using the Caliper GX LabChip GX/HT DNA High Sensitivity Kit (Cat no: CLS760672, PerkinElmer). Before sequencing, the libraries were normalized and pooled. Sequencing was performed on an Illumina MiSeq v3–600 flowcell with a 301–10–10–301 read setup, ensuring high-quality data generation.

#### 2.2.4 16S rRNA profiling

Analysis of 16S rRNA sequencing data was performed using the Nextflow pipeline ampliseq v2.2.0^1^. Briefly, raw sequencing reads were quality-checked initially using FastQC (12), followed by trimming of primer sequences from the reads using cutadapt v3.4 (13). Sequencing reads were denoised, dereplicated, and filtered for chimeric sequences using DADA2 (14). Denoised paired-end reads were truncated from position 279 (forward) and 229 (reverse), whereas all other reads shorter than 50 bp were removed. The truncated sequences were merged with minimum 12 bp overlap, resulting in a total of 4,837 amplicon sequence variants (ASVs) of which 645 unassigned ASVs were removed before for further analysis. These ASVs were taxonomically classified from phylum to species level using the SILVA v138 prokaryotic SSU database (15) by applying Naive Bayes classifier implemented in QIIME 2 (16), trained on the pre-processed database. Following taxonomic classification of ASVs classified as Mitochondria or Chloroplast were removed.

### 2.3 Statistical analyses

Data analysis was performed using R statistical software (R Core Team, 2022; v 4.2.2). Alpha diversity significance for bacterial diversity, richness within the samples and species evenness (Pielou) was determined using Kruskal-Wallis test. Beta diversity significance was determined using overall and pairwise PERMANOVA tests with a Bonferroni corrected *p*-value < 0.05. For the microbiome data, differential abundance of ASVs was calculated with DESeq2 v 1.38.3 (17) using Wald test with a corrected *p* < 0.05. Pearson correlations were calculated between ASVs. Correlations with p-value < 0.05 were considered significant. The results of bacterial identification using MALDI-TOF MS are presented descriptively, highlighting species distribution across breeds, collection times, and individual bulls.

## 3 Results

### 3.1 MALDI-TOF MS

There were variation in bacterial appearances between ejaculates and individual bulls (Table 1). In total, 33 bacterial species were identified by MALDI TOF from 20 bull and 60 ejaculates, while 88 bacteria colonies could not be identified. Only nine bacterial species were consistently detected in semen samples collected at all three time points: *Bacillus licheniformis, Bacillus pumilus, Corynebacterium xerosis, Enterobacter cloacae, Escherichia coli, Proteus mirabilis, Serratia rubidaea, Staphylococcus chromogenes* and *Staphylococcus sciuri*. Furthermore, of the 33 identified bacterial species, six were found exclusively in semen samples from VH bulls, 12 were identified in semen from VR bulls, and 15 were isolated from semen samples of both breeds (Table 1). Finally, three, seven and five bacteria species were identified in only one occasion, i.e., in first, second and third ejaculates, respectively. On the individual bull level, different *Bacillus* spp., *Proteus* spp., and *Staphylococcus* spp., were identified in the majority of bull semen samples and all collection-time. However, *Proteus* spp., was isolated only from VR bulls, while *Bacillus* spp. and *Staphylococcus* spp. were present in the samples from both breeds.

**Table 1:** Bacterial species in semen samples from 20 individual bulls (1–20) of Viking Holstein (VH) and Viking Red (VR) collected at three samplings occasions and identified by MALDI-TOF MS.

Bacterial species	1st ejaculate	2nd ejaculate	3rd ejaculate	Breed
*Alkalihalobacillus clausii^§^*		2		VH
*Actinobacillus seminis^§^*			3	VH
*Bacillus cereus*	15		1, 7, 14	VH/VR
Bacillus licheniformis^*^	6, 9	1, 3, 9, 14	5, 6, 8, 9, 18	VH/VR
Bacillus pumilus^*^	2, 5, 9, 11, 19	2, 5, 8, 14, 17	2, 5, 7, 8, 9, 16, 17	VH/VR
*Bacillus subtilis*		1	10	VH/VR
*Brevibacillus parabrevis*		1	10	VH/VR
*Corynebacterium cystitidis*		5	8	VH/VR
*Corynebacterium freneyi^§^*		10		VR
Corynebacterium xerosis^*^	3	3, 17	1	VH
*Enterobacter bugandensis*		8	6	VH/VR
Enterobacter cloacae^*^	3, 11	8	8, 11	VH/VR
*Enterobacter ludwigii*		7	6	VH
Escherichia coli^*^	9	18	9	VR
*Histophilus somni^§^*			6, 18	VH/VR
*Micrococcus lylae^§^*		3		VH
*Micrococcus luteus*	2	1		VH
*Neisseria elongata^§^*			10	VR
*Neisseria subflava^§^*			8	VR
*Proteus hauseri^§^*	16			VR
Proteus mirabilis^*^	13, 20	20	15, 20	VR
*Proteus vulgaris*	16		11	VR
*Serratia liquefaciens*	5	9		VH/VR
Serratia rubidaea^*^	1, 19	1, 9	1, 19	VH/VR
Staphylococcus chromogenes^*^	1, 3, 5, 9	1, 3, 5, 8	5, 10	VH/VR
*Staphylococcus epidermidis*	6, 12			VH/VR
*Staphylococcus pasteuri^§^*		8		VR
Staphylococcus sciuri^*^	9, 15	2, 7	2, 6, 12, 14	VH/VR
*Staphylococcus warneri^§^*	2, 4, 6, 12			VH/VR
*Streptococcus dysgalactiae^§^*		17		VR
*Streptococcus oralis^§^*			12	VR
*Streptococcus uberis^§^*		18		VR
*Trueperella pyogenes^§^*		17		VR

* Bacterial species identified across all ejaculates, § represent bacterial species that were identified on only one occasion.

### 3.2 16S sequencing

**Figure 1:**
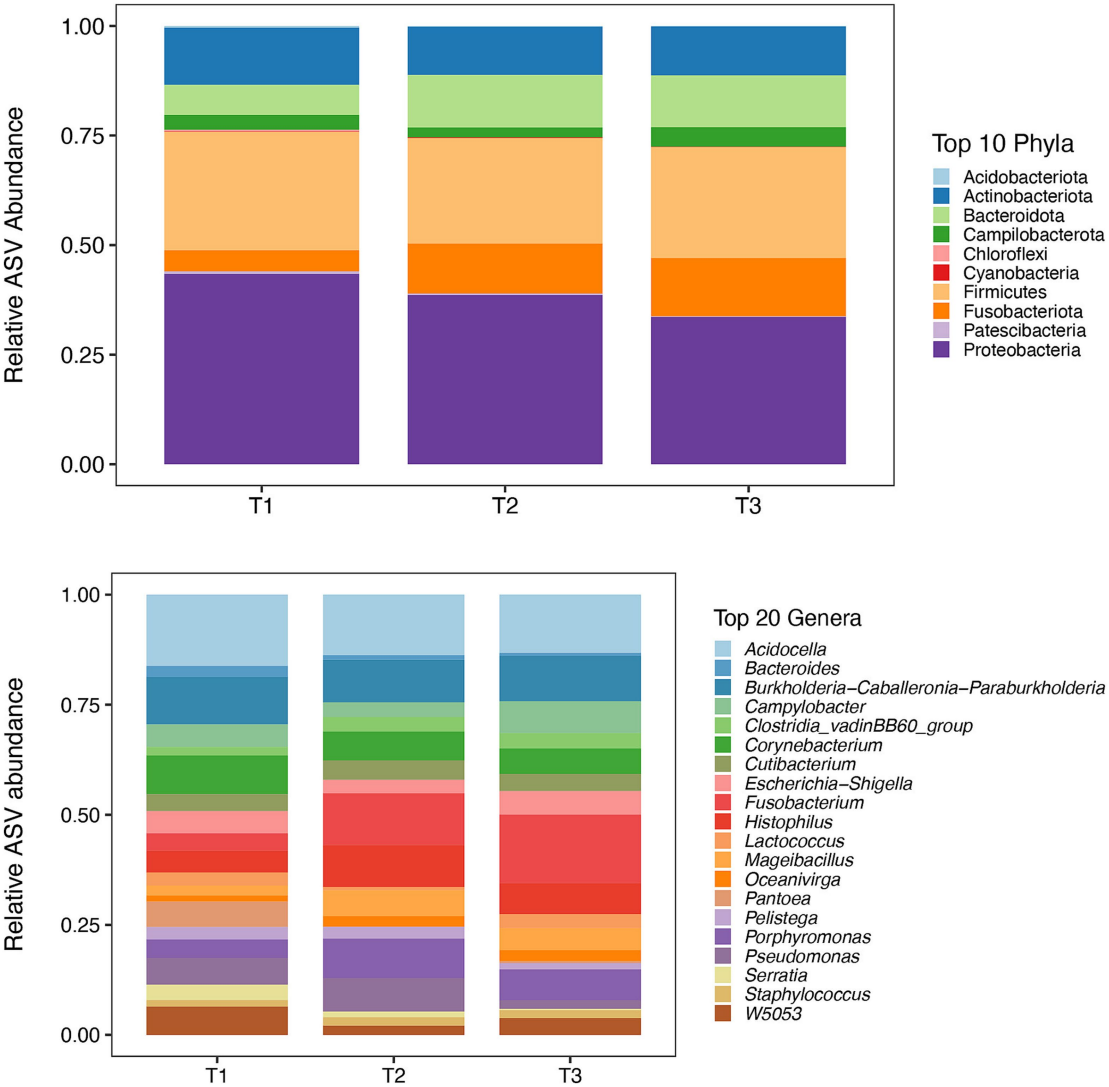
Mean relative abundance of top 10 phyla **(Top)** and 20 most abundant genera **(Bottom)** for each sample time points (T1, T2, and T3) identified by 16S rRNA sequencing. *: T1 – first ejaculate 96 h after previous collection, T2 – second ejaculates 48 h after the first, T3 – third ejaculates 24 h after the second.

A total of 4,837 ASVs was identified across all the samples. In total, 23 phyla and 402 genera were identified in the 60 bull semen samples from three different ejaculates, of which Top 10 phyla and Top 20 genera are present in Figure 1.

**Figure 2:**
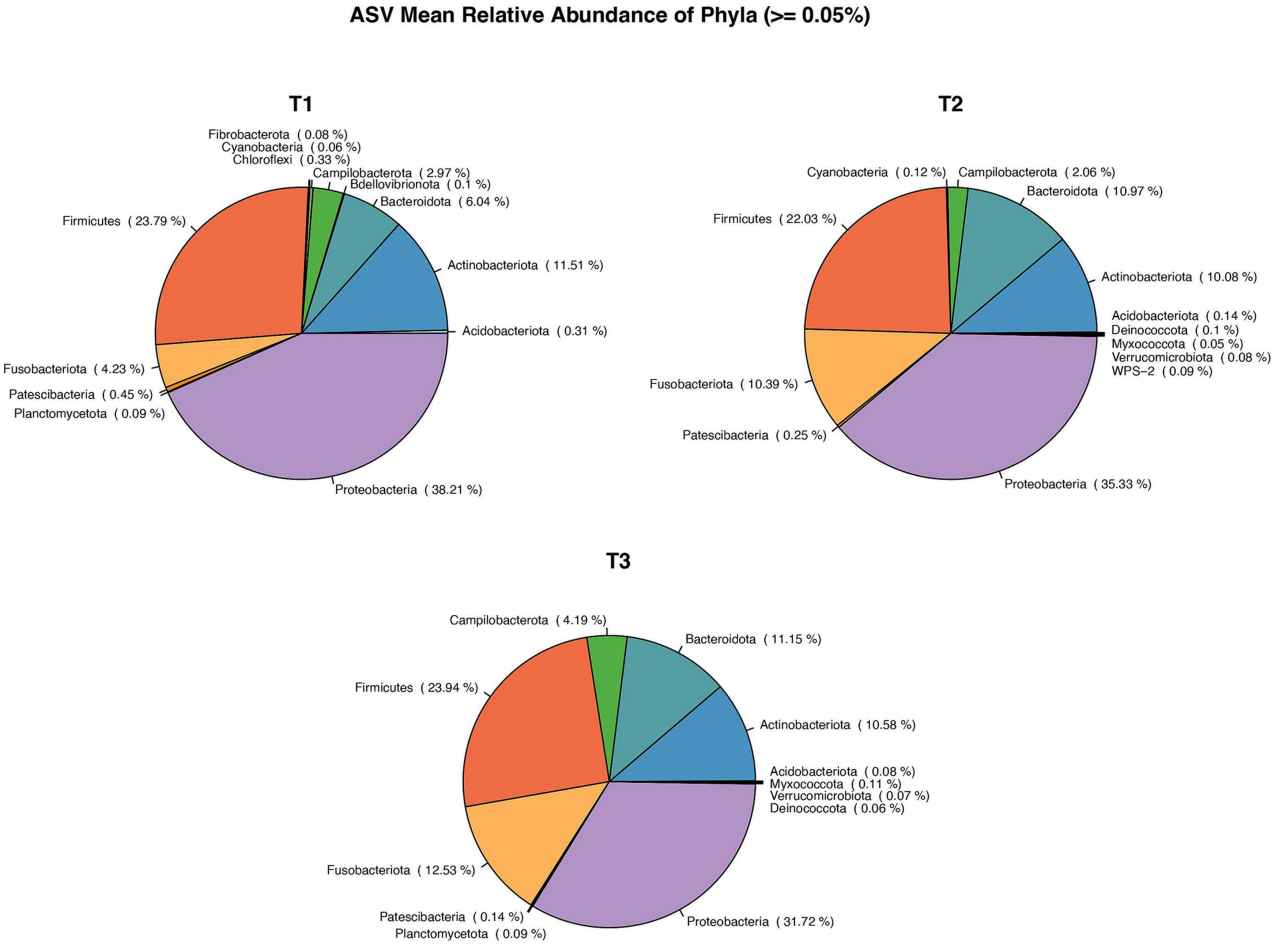
Pie chart showing Mean relative abundance of Phyla between the sample time points: T1, T2, T3. *: T1 – first ejaculate 96 h after previous collection, T2 – second ejaculates 48 h after the first, T3 - third ejaculates 24 h after the second.

In first (T1), second (T2) and third (T3) timepoints, 16, 21, 21 phyla and 228, 254, 306 genera, respectively, were identified. The mean relative abundance of Phyla with a prevalence of ≥0.5% is presented in Figure 2.

**Figure 3:**
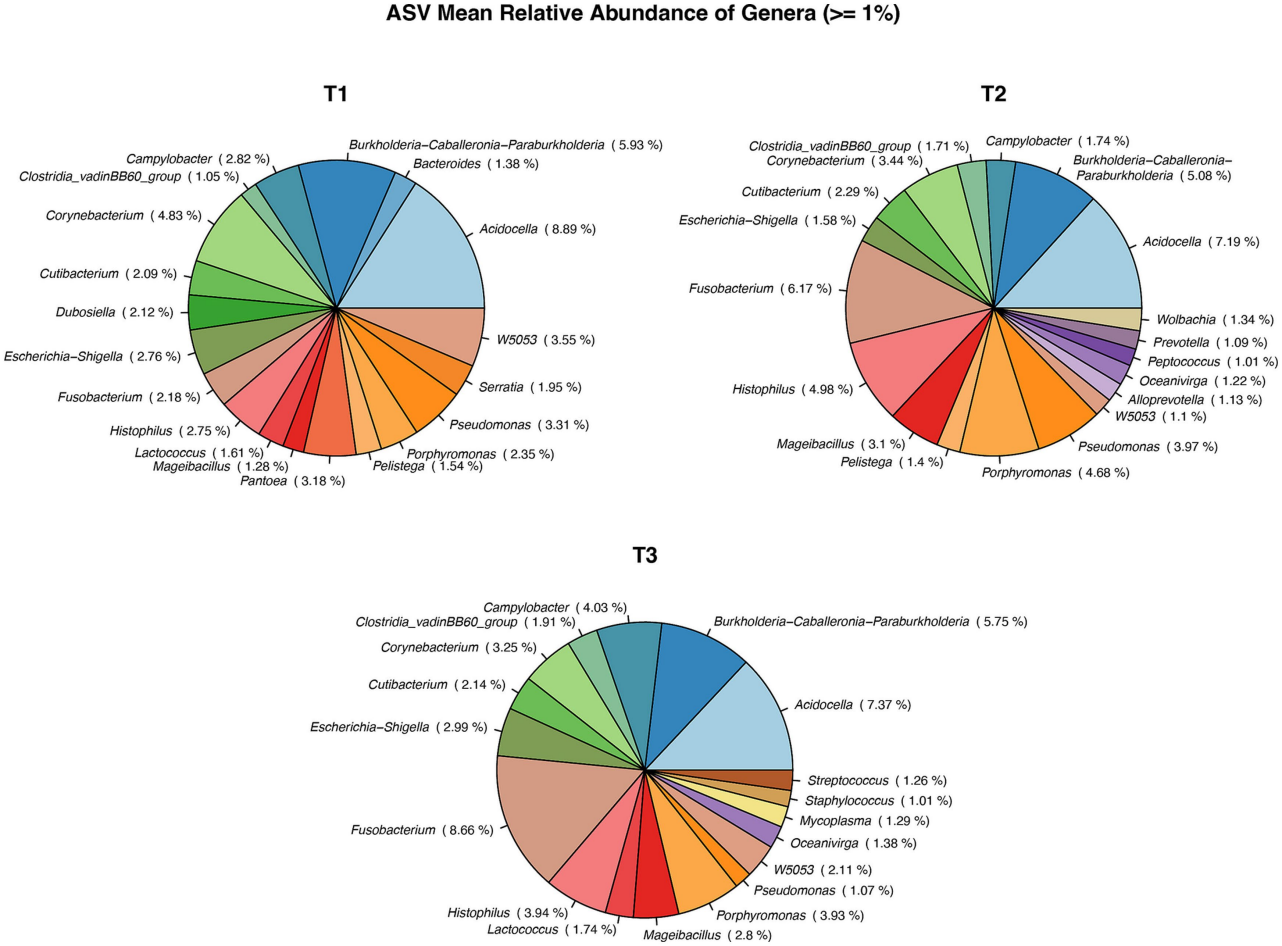
Pie chart showing Mean relative abundance of Genera between the sample time points: T1, T2, T3. *: T1 – first ejaculate 96 h after previous collection, T2 – second ejaculates 48 h after the first, T3 – third ejaculates 24 h after the second.

The number of bacterial genera differing by >1% in relative abundance was similar for all collection times (Figure 3). In total, 14 genera were present at all collection times, three were present at two collection times, and three, four, and three genera were present on only one occasion: T1 (*Bacteroides, Serrata, Pantoea*), T2 (*Wolbachia, Prevotella, Peptococcus, Alloprevotella*), and T3 (*Streptococcus, Staphyloccocus, Mycoplasma*), respectively. All 402 genera identified at different collection times are presented in Supplementary Table 2.

There was no significant difference (*p* > 0.05) in seminal bacterial alpha diversity (Shannon Index), community evenness (Pielou, data not shown) between the three time points (T1, T2, T3). However, Alpha diversity (Shannon) between the bull samples across all time points did show a trend towards significance *p* = 0.07 and so did the alpha diversity (Observed) *p* = 0.07 (Supplementary Figure 1). Shannon diversity index showed a moderate effect size (ε^2^ = 0.29), indicating that nearly 29% of variation in alpha diversity was influenced by individual bull. Nevertheless, alpha diversity between breeds was significant for both Shannon (*p* = 0.01) and Observed metrics (*p* = 0.005; Supplementary Figure 2). However, breed accounted for only a small effect on alpha diversity (ε^2^ = 0.29). Bull semen community structure (beta diversity) did not show a significant difference (*p* > 0.05) in Bray Curtis measurements (Supplementary Figure 3) between time points. However, a significant difference in beta diversity was observed between breeds, where VH samples formed a cluster whereas VR samples had a more diversified microbiome (Supplementary Figure 4).

Amplicon sequence variants (ASV) and number of species identified by MALDI-TOF, which overlap between semen collection-time points and bull breed, are presented in Table 2.

**Table 2:** Number of identified bacterial isolates (MALDI TOF MS) and AVSs (16S Sequencing) across different semen collection time points and bull breed.

	MALDI TOF MS	16S sequencing
Time		
T1	3	1,285
T2	7	988
T3	5	1,157
T1/T2	2	92
T1/T3	4	125
T2/T3	0	183
T1/T2/T3	9	334
Breed		
VH	6	1,027
VR	12	2,607
VH/VR	15	339

*T1 – first ejaculate 96 h after previous collection, T2 – second ejaculates were collected 48 h after the first, T3 – third ejaculates were collected 24 h after the second. VH – Viking Holstein, VR – Viking red.

**Figure 4:**
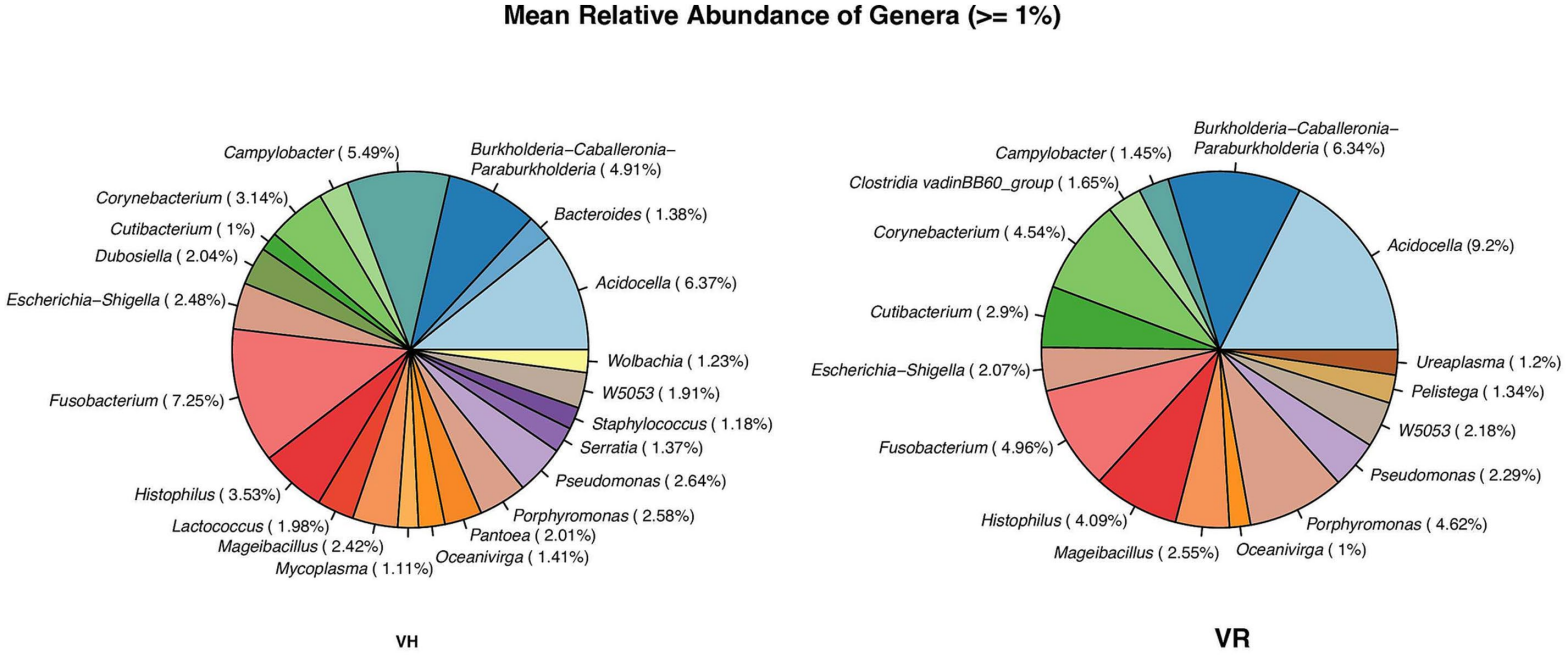
Differences in distribution type of bacteria differing by >1% in relative abundance between breeds, VH – Viking Holstein, VR – Viking red.

The number of phyla and genera differed between breeds with 13 and 160 identified for VH, and 23 and 377 for VR, respectively. There was no difference in mean relative abundance of phyla (> = 0.05%) between breeds. In contrast, there were differences in the number and type of bacteria differing by > 1% in relative abundance between breeds (Figure 4).

In total, 21 genera were identified, with *Bacterioides, Wolbachia, Staphylococcus, Serratia, Pantoea, Mycoplasma, Lactococcus* and *Dubosiella* appearing only in the VH group. In VR group, 16 genera were identified, with *Ureoplasma* and *Clostridia* only in this group.

**Figure 5:**
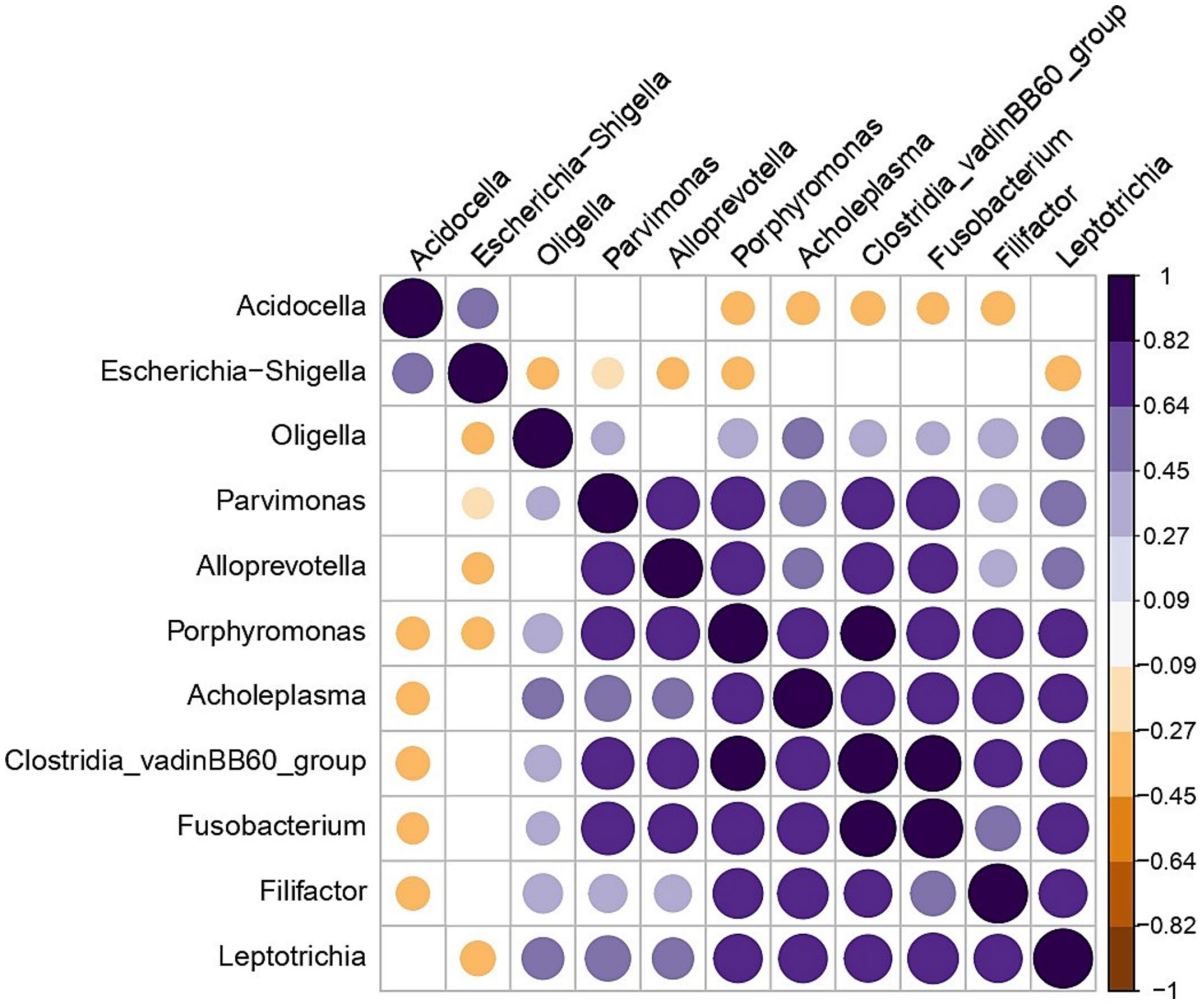
Correlation plot showing genera that have a negative correlation with each other in all the bull samples. Significant correlation (*p* < 0.05) is presented by blue dots for positive and brown dots for negative correlations. Blank cells indicate non-significant correlations.

The genera *Acidocella* and *Escherichia* exhibited a negative correlation with the majority of bacterial taxa, while displaying a slight positive correlation with each other (Figure 5- plotted from 16S rRNA sequencing data).

**Figure 6:**
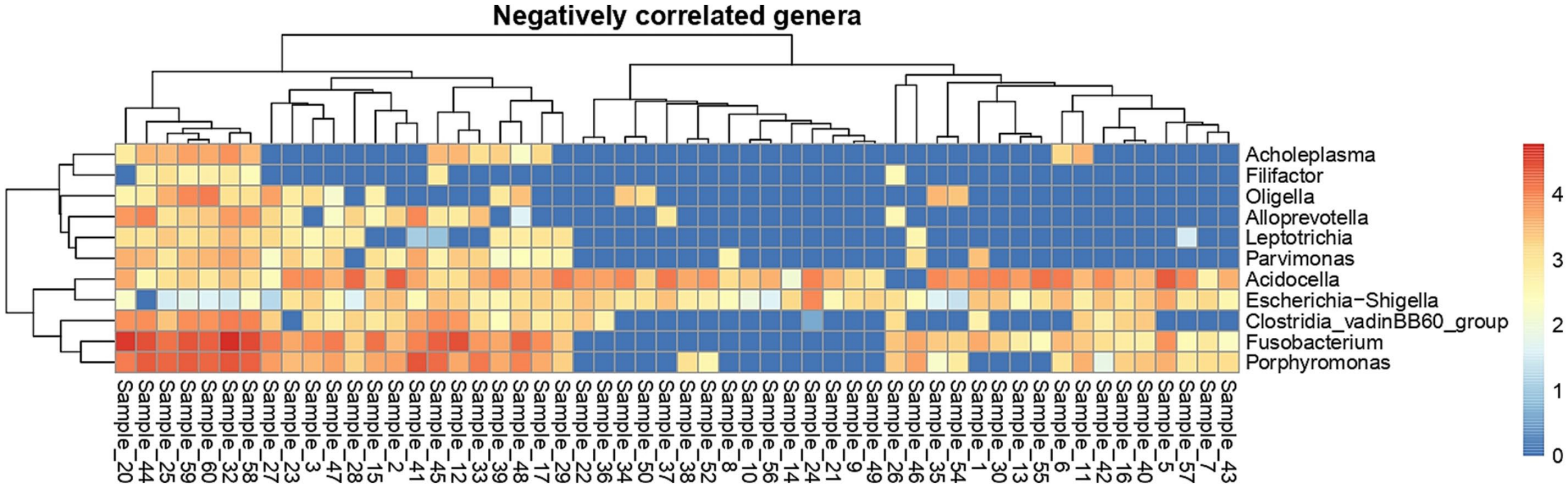
Heat map showing sample-wise abundance of negatively correlated genera. The color in the heat map cells indicates log10 counts of every genus in each sample.

At the individual sample level (Figure 6 - plotted from 16S rRNA sequencing data), *Acidocella* was present in all semen samples except two samples.

## 4 Discussion

This study investigated the variations in bacterial occurrence across ejaculates from individual bulls, with additional emphasis on potential breed-associated differences in seminal microbiota. The combined use of 16S rRNA sequencing and culture with identification by MALDI-TOF MS provided a robust approach for bacterial identification, allowing a detailed characterization of seminal microbiota composition. Additionally, there is a lack of knowledge regarding differences in bacterial occurrence based on the frequency of semen collection. While previous studies have utilized MALDI-TOF MS for bacterial identification after culture, their focus has primarily been on the detection of bacterial pathogens (18), the identification of difficult-to-characterize bacterial strains (19), and the assessment of clinically relevant anaerobic bacteria (20), often for the critical evaluation of discrepant results. In recent years, and even over the past decades, MALDI-TOF MS and metagenomic analyses has been applied separately, to analyze the semen microbiota of various species, including bulls (9, 11, 21), stallions (22, 23), boar (24) and roosters (25). Furthermore, these techniques, in combination, have been employed for species-level identification of individual bacterial strains, *Lactobacillus* spp. in vaginal (26) and *Streptococcus* spp. in oral (27) samples.

Several studies have provided an in-depth examination of the bacterial composition of bull semen microbiota by 16S sequencing, as well as its potential impact on sperm quality (28) and fertility (9). Additionally, various factors influencing seminal microbiota have been investigated, including seasonal variations (21), age of male (29) and feeding practices (6).

At the species level (identified by MALDI TOF MS), our results revealed noticeable variability in bacterial composition among ejaculates collected at different time points. Despite this variation, nine bacterial species, including *Bacillus licheniformis, Escherichia coli,* and *Staphylococcus chromogenes*, were consistently present across all ejaculates. These ubiquitous bacteria may form a stable core microbiome that remains consistent over time. However, unique bacterial species were identified in specific ejaculate collection time points. For example, *Bacteroides* and *Serratia* were found exclusively in the first ejaculate, whereas *Wolbachia* and *Prevotella* appeared only in the second. This suggests that certain bacteria may transiently colonize the reproductive tract but are subsequently competed out, or that ejaculate-specific factors may influence microbial composition.

Despite the variability in bacterial species identified by MALDI-TOF MS, the 16S rRNA gene sequencing at the genus level showed no significant differences in alpha diversity indices across the three time points. Beta diversity analysis also indicated no significant shifts in microbial community structure between time points, suggesting a relatively stable microbiota at the community level despite fluctuations in specific taxa. In a previous study conducted by the same group of authors (21), several factors contributing to variations in bull semen microbiota at the genus level were discussed. These included temporal changes in microbiota composition within living animals, as well as differences arising from sample type—specifically, raw ejaculates compared to commercially processed semen, where handling at bull stations may introduce cross-contamination between samples or from personnel. Additionally, geographical influences were considered, as animals housed in different countries or even distinct facilities may develop unique microbiomes (30, 31). The findings of the present study further suggest that breed-related differences exist, as both breeds were maintained under identical conditions on the same premises, and semen was collected on the same days by the same personnel. While both VH and VR breeds shared several taxa, the number of genera was substantially higher in the VR group, which also exhibited greater microbial diversity. However, clear differences in microbiota composition were observed between bull breeds. For instance, identified by MALDI-TOF MS *Proteus* spp. were exclusive to VR bulls identified by MALDI-TOF MS, while *Bacillus* spp. and *Staphylococcus* spp. were common to both breeds. Interestingly in the results of 16S sequencing, distinct genera such as *Ureaplasma* and *Clostridia* were identified only in the VR group, whereas *Bacteroides* and *Mycoplasma* were unique to VH bulls. Alpha diversity metrics showed significantly higher diversity in VR bulls compared to VH bulls, suggesting that breed-specific factors, such as genetic or physiological differences, may influence seminal microbial composition. Additionally, beta diversity analysis revealed that VH bulls exhibited a more clustered microbiota, while VR bulls harbored a more heterogeneous community structure, indicating greater variability within the VR group. Notably, the genera *Acidocella* and *Escherichia* demonstrated an negative correlation with the majority of bacterial taxa but were positively correlated with each other. The widespread presence of *Acidocella* across samples, coupled with its negative association with other taxa, suggests it may play a competitive or regulatory role in the seminal microbial ecosystem. Further assessing of these interactions is needed to better understand their functional significance.

Due to lack of studies regarding differences in semen bacterial microbiota identified by 16S rRNA sequencing between bull breeds housed under the same conditions, we are unable to directly compare our findings with those of other researchers. However, previous research has demonstrated seasonal differences in the bacterial microbiota of bull semen collected in different season of the year (21), as well as in boar semen microbiota between boars of different age (29). In the boar study (29), microbiota analysis using the 16S rRNA technique revealed that semen from older individuals exhibited reduced alpha- and beta-diversity compared to younger boars. Furthermore, specific bacterial taxa were identified as biomarkers for semen quality in different age groups. The *Streptococcus* spp., including *Streptococcus gallolyticus* subsp. *macedonicus*, were associated with semen from younger breeding boars, whereas *Bacteroides pyogenes* was identified as a biomarker for semen from older breeding boars (29). Additionally, semen from older boars exhibited a higher abundance of *Aerococcus, Gallicola, Ulvibacter*, and *Proteiniphilum* compared to younger boars. Spearman correlation analysis indicated that these four bacterial genera were negatively correlated with semen quality (29). These findings highlight the potential influence of host factors such as age and breed on seminal microbiota composition.

In the present study on bull semen, bacteria from the *Mycoplasma* genus were identified with a relative abundance of ≥1%, exclusively in the VH group. The most significant species within this genus is *M. bovis,* a bacterium that causes substantial economic losses and adversely affects animal welfare in the cattle industry (32). Clinical manifestations of infection vary, with respiratory disease, mastitis, and joint infections being the most frequently observed clinical signs (33). In the study of Haapala et al. (34), bull semen was described as the source of *M. bovis* into two closed, biosecure dairy herds via contaminated semen used in artificial insemination (AI), which subsequently resulted in mastitis outbreaks in both herds. Given that *M. bovis* is challenging to culture and identify due to its slow growth rate and specific environmental requirements, its detection in semen samples underscores the need for sensitive and rapid molecular diagnostic methods, such as PCR (35). Furthermore, in regions with a low prevalence of *M. bovis*, identifying potential transmission routes from less common sources such as semen samples is crucial, as the pathogen circulates less frequently than in high-prevalence populations (34). The presence of *M. bovis* in bull semen suggests that artificial insemination may serve as a potential transmission route, emphasizing the importance of routine screening in breeding programs. This study is particularly important as it highlights the presence of *Mycoplasma* in bull semen at a relative abundance of >1% within the VH group and in samples collected during the third collection time (T3). The fact that *Mycoplasma* appeared in the T3 collection further suggests possible fluctuations in its presence over time, which could be influenced by factors such as host immune responses or bacterial shedding patterns. These findings highlight the necessity of continued surveillance and improved biosecurity measures to prevent the spread of *M. bovis* in cattle populations. Since *M. bovis* is not typically included in routine screening protocols for breeding bulls, undiagnosed carriers may continuously disseminate the bacteria, making eradication efforts more complex.

The present study highlights collection-time and breed-associated differences in the seminal microbiota of bulls. The presence of a core microbiome across ejaculates, despite collection-time variability, underscores the resilience of certain bacterial taxa. However, the observed breed-specific differences in bacterial diversity and composition emphasize the potential influence of host factors on seminal microbiota. While no significant changes in microbial diversity were observed between ejaculates, the distinct bacterial profiles identified at specific collection times may have implications for understanding bacterial dynamics in the reproductive tract. These findings contribute to the growing body of knowledge on the bovine seminal microbiome and provide a foundation for future research exploring its role in bull fertility and reproductive performance.

Other matters that need to be discussed are limitations in methods for bacterial identification described in this study. Identification by MALDI-TOF MS depends on a reference library. If a bacterial species is not represented in the library, it cannot be accurately identified, leading to inability to assign a species-level identification. In our study, 88 bacterial species could not be identified using MALDI, which is more than 2.5 times the number of identified species. This represents a significant challenge, as some of unidentified bacteria may play crucial roles in semen quality, fertility, and health. This limitation is particularly relevant for less-characterized such as non-pathogenic environmental strains that may be present in the seminal microbiota but remain unidentified due to limitations in the reference library. Unlike MALDI-TOF MS, 16S sequencing does not require bacterial culture and allows for the identification of a broad range of bacteria within a sample. However, since 16S sequencing detects bacterial DNA rather than “live bacteria,” it does not confirm bacterial viability or directly link specific bacteria to sperm quality and/or fertility issues, although it does provide insights into microbial presence. A promising complementary method to address this issue is viability PCR, which uses intercalating dyes to selectively inhibit amplification of DNA from dead cells. While this technique offers theoretical advantages, it also comes with notable limitations and technical challenges. As reviewed by Codony et al. (36), despite continuous methodological improvements since its introduction, the consistent and reliable discrimination of viable bacteria remains difficult in many practical applications.

Correlations of *Acidocella* and *Escherichia* with other bacteria suggests that their presence may be associated with a competitive exclusion of other bacteria, potentially influencing the overall semen microbiota composition. In a previous study of bull semen microbiota across different seasons (21), *Acidocella* was present in the majority of samples and, along with *Escherichia*, represented the genus with the highest relative ASV abundance. It also showed a similar distribution in winter and spring compared to summer. The implications of these findings remain unclear but may support further investigation into their role in microbial dynamics within bull semen. The study found breed-specific differences in semen microbiota, with certain bacterial species and genera being unique to each breed. Since both breeds were housed under the same conditions, these differences likely emerge from intrinsic factors such as genetics, immune response, or breed-specific physiology. These findings suggest that husbandry practices, including hygiene measures and semen collection routines, may need to be tailored to specific breeds to minimize bacterial contamination and ensure semen quality. The results did not indicate a clear “best” abstinence time, as no significant differences in seminal bacterial alpha diversity were observed between collection time points (T1, T2, and T3). In study of Taaffe et al. (37), more frequent semen collection in young dairy bulls enhances semen production and fertility rates in field applications. Nevertheless, previous studies reported shorter collection times than observed in our research, indicating variations in collection protocols or bull responses that influencing semen quality, ejaculate characteristics, and collection efficiency, which may be also attributed to differences in semen microbiota. However, some bacterial species appeared only at specific collection times, such as *Mycoplasma* in T3, which could suggest that semen microbiota may be influenced by the frequency of collection. Further research is needed to determine whether adjusting abstinence periods could optimize semen microbiota for fertility outcomes.

## 5 Conclusion

The seminal microbiota of bulls exhibits both temporal variability and breed-specific differences. The identification of a core microbiome and key taxa unique to specific breeds or collection times suggests that the seminal microbiota is shaped by a complex interplay of environmental, physiological, and host factors. Understanding these dynamics could inform strategies to optimize reproductive health and semen quality in livestock breeding programs. By integrating both methodologies, our study provides a comprehensive characterization of the seminal microbiota in bulls, offering novel insights into microbial dynamics across different semen collection-time points and between breeds.

## Data Availability

The original contributions presented in the study are publicly available. This data can be found here: European Nucleotide Archive (ENA), accession PRJEB96305.

## Footnotes

^1^ https://github.com/nf-core/ampliseq

## References

[1] Goularte K Madeira E Ferreira C Duval E Vieira A Mondadori R. Hazard analysis and critical control points system for a bull semen production Centre. Reprod Domest Anim. (2015) 50:972–9. doi: 10.1111/rda.12617, PMID: 26477334

[2] Shukla M. Applied veterinary andrology and frozen semen technology. Pitam Pura, New Delhi: New India Publishing Agency (2011).

[3] EUR-Lex. Commission delegated regulation (Eu) 2021/880. (2021)

[4] Teale C Moulin G. Prudent use guidelines: a review of existing veterinary guidelines. Rev Sci Tech Off Int Epiz. (2012) 31:343–54. doi: 10.20506/rst.31.1.2119, PMID: 22849288

[5] Prince P Almquist J Reid J. Bacteriological studies of bovine semen. II. The incidence of specific types of bacteria and the relation to fertility. J Dairy Sci. (1949) 32:849–55. doi: 10.3168/jds.S0022-0302(49)92126-8

[6] Webb EM Holman DB Schmidt KN Crouse MS Dahlen CR Cushman RA. A longitudinal characterization of the seminal microbiota and antibiotic resistance in yearling beef bulls subjected to different rates of gain. Microbiol Spectr. (2023) 11:e05180–22. doi: 10.1128/spectrum.05180-22 36916922 PMC10100376

[7] Gupta D Sarkar A Pal Y Suthar V Chawade A Kushwaha SK. Bovine reproductive tract and microbiome dynamics: current knowledge, challenges, and its potential to enhance fertility in dairy cows. Front Microbiolomes. (2024) 3:1473076. doi: 10.3389/frmbi.2024.1473076 PMC12993595 41853553

[8] Kilama J Dahlen CR Reynolds LP Amat S. Contribution of the seminal microbiome to paternal programming. Biol Reprod. (2024) 111:242–68. doi: 10.1093/biolre/ioae068, PMID: 38696371 PMC11327320

[9] Cojkic A Niazi A Guo Y Hallap T Padrik P Morrell JM. Identification of bull semen microbiome by 16s sequencing and possible relationships with fertility. Microorganisms. (2021) 9 20211125. doi: 10.3390/microorganisms9122431, PMID: 34946031 PMC8705814

[10] Croxatto A Prod'hom G Greub G. Applications of Maldi-Tof mass spectrometry in clinical diagnostic microbiology. FEMS Microbiol Rev. (2012) 36:380–407. doi: 10.1111/j.1574-6976.2011.00298.x, PMID: 22092265

[11] Cojkic A Hansson I Johannisson A Axner E Morrell JM. Single layer centrifugation as a method for bacterial reduction in bull semen for assisted reproduction. Vet Res Commun. (2024) 48:39–48. doi: 10.1007/s11259-023-10178-y, PMID: 37479850 PMC10811171

[12] Andrews S. Fastqc: A quality control tool for high throughput sequence data. Cambridge, United Kingdom: Babraham Bioinformatics, Babraham Institute (2010).

[13] Martin M. Cutadapt removes adapter sequences from high-throughput sequencing reads. EMBnet J. (2011) 17:10–2. doi: 10.14806/ej.17.1.200

[14] Callahan BJ McMurdie PJ Rosen MJ Han AW Johnson AJA Holmes SP. Dada2: high-resolution sample inference from Illumina amplicon data. Nat Methods. (2016) 13:581–3. doi: 10.1038/nmeth.3869, PMID: 27214047 PMC4927377

[15] Quast C Pruesse E Yilmaz P Gerken J Schweer T Yarza P. The Silva ribosomal Rna gene database project: improved data processing and web-based tools. Nucleic Acids Res. (2012) 41:D590–6. doi: 10.1093/nar/gks1219, PMID: 23193283 PMC3531112

[16] Bolyen E Rideout JR Dillon MR Bokulich NA Abnet CC Al-Ghalith GA. Reproducible, interactive, scalable and extensible microbiome data science using Qiime 2. Nat Biotechnol. (2019) 37:852–7. doi: 10.1038/s41587-019-0209-9, PMID: 31341288 PMC7015180

[17] Love MI Huber W Anders S. Moderated estimation of fold change and dispersion for Rna-Seq data with Deseq2. Genome Biol. (2014) 15:1–21. doi: 10.1186/s13059-014-0550-8, PMID: 25516281 PMC4302049

[18] Schröttner P Gunzer F Schüppel J Rudolph WW. Identification of rare bacterial pathogens by 16s Rrna gene sequencing and Maldi-Tof Ms. J Visualized Experiments. (2016) 113:53176. doi: 10.3791/53176-v, PMID: 27500532 PMC4993432

[19] Bizzini A Jaton K Romo D Bille J Prod'hom G Greub G. Matrix-assisted laser desorption ionization–time of flight mass spectrometry as an alternative to 16s rRNA gene sequencing for identification of difficult-to-identify bacterial strains. J Clin Microbiol. (2011) 49:693–6. doi: 10.1128/JCM.01463-10, PMID: 21106794 PMC3043501

[20] Cobo F Pérez-Carrasco V Martín-Hita L García-Salcedo JA Navarro-Marí JM. Comparative evaluation of Maldi-Tof Ms and 16s Rrna gene sequencing for the identification of clinically relevant anaerobic bacteria. Critical evaluation of discrepant results. Anaerobe. (2023) 82:102754. doi: 10.1016/j.anaerobe.2023.102754, PMID: 37321445

[21] Cojkic A Niazi A Morrell JM. Metagenomic identification of bull semen microbiota in different seasons. Anim Reprod Sci. (2024) 268:107569. doi: 10.1016/j.anireprosci.2024.107569, PMID: 39098060

[22] Malaluang P Niazi A Guo Y Nagel C Guimaraes T Rocha A. Bacterial diversity in semen from stallions in three European countries evaluated by 16s sequencing. Vet Res Commun. (2024) 48:1409–21. doi: 10.1007/s11259-024-10321-3, PMID: 38305959 PMC11147884

[23] Al-Kass Z Guo Y Pettersson OV Niazi A Morrell J. Metagenomic analysis of bacteria in stallion semen. Anim Reprod Sci. (2020) 221:106568. doi: 10.1016/j.anireprosci.2020.106568 32861118

[24] McAnally BE Smith MS Wiegert JG Palanisamy V Chitlapilly Dass S Poole RK. Characterization of boar semen microbiome and association with sperm quality parameters. J Anim Sci. (2023) 101:skad243. doi: 10.1093/jas/skad243, PMID: 37464945 PMC10393202

[25] Tvrdá E Petrovičová M Benko F Ďuračka M Kováč J Slanina T. Seminal Bacterioflora of two rooster lines: characterization, antibiotic resistance patterns and possible impact on semen quality. Antibiotics. (2023) 12:336. doi: 10.3390/antibiotics12020336, PMID: 36830247 PMC9952488

[26] Anderson AC Sanunu M Schneider C Clad A Karygianni L Hellwig E. Rapid species-level identification of vaginal and oral lactobacilli using MALDI-TOF MS analysis and 16S rDNA sequencing. BMC Microbiol. (2014) 14:1–9. doi: 10.1186/s12866-014-0312-5 PMC4272787 25495549

[27] Yıldız SS Kaşkatepe B Altınok S Cetin M Karagöz A Savaş S. Comparison of Maldi-Tof and 16s Rrna methods in identification of Viridans group streptococci. Mikrobiyol Bul. (2017) 51:1–9. doi: 10.5578/mb.46504 28283005

[28] Ďuračka M Belić L Tokárová K Žiarovská J Kačániová M Lukáč N. Bacterial communities in bovine ejaculates and their impact on the semen quality. Syst Biol Reprod Med. (2021) 67:438–49. doi: 10.1080/19396368.2021.1958028, PMID: 34445906

[29] Li D Xu Y Wang M Fang S Li SH Cui Y. Differences of semen microbiota among breeding boars with different reproductive ages. J Anim Sci. (2023) 101:skad247. doi: 10.1093/jas/skad247, PMID: 37478469 PMC10424712

[30] Sannat C Nair A Sahu SB Sahasrabudhe SA Rawat N Shende RK. Effect of season on bacterial load in semen of different breeds of cattle. J Anim Res. (2016) 6:651–6. doi: 10.5958/2277-940X.2016.00077.2

[31] Mocé ML Esteve IC Pérez-Fuentes S Gómez EA Mocé E. Microbiota in goat buck ejaculates differs between breeding and non-breeding seasons. Front Veter Sci. (2022) 9:867671. doi: 10.3389/fvets.2022.867671, PMID: 35647092 PMC9136232

[32] Nicholas R Ayling R. *Mycoplasma bovis*: disease, diagnosis, and control. Res Vet Sci. (2003) 74:105–12. doi: 10.1016/s0034-5288(02)00155-8, PMID: 12589733

[33] Byrne AW Barrett D Breslin P Fanning J Casey M Madden JM. Bovine tuberculosis in Youngstock cattle: a narrative review. Front Veter Sci. (2022) 9:1000124. doi: 10.3389/fvets.2022.1000124, PMID: 36213413 PMC9540495

[34] Haapala V Pohjanvirta T Vähänikkilä N Halkilahti J Simonen H Pelkonen S. Semen as a source of *Mycoplasma bovis* mastitis in dairy herds. Vet Microbiol. (2018) 216:60–6. doi: 10.1016/j.vetmic.2018.02.005, PMID: 29519526

[35] Hazelton M Morton J Bosward K Sheehy P Parker A Dwyer C. Isolation of *Mycoplasma* spp. and serological responses in bulls prior to and following their introduction into *Mycoplasma bovis*-infected dairy herds. J Dairy Sci. (2018) 101:7412–24. doi: 10.3168/jds.2018-14457, PMID: 29753469

[36] Codony F Dinh-Thanh M Agustí G. Key factors for removing Bias in viability Pcr-based methods: a review. Curr Microbiol. (2020) 77:682–7. doi: 10.1007/s00284-019-01829-y, PMID: 31811375

[37] Taaffe P O'Meara C Stiavnicka M Byrne C Eivers B Lonergan P. Increasing the frequency of ejaculate collection in young dairy bulls increases semen production and field fertility. Theriogenology. (2022) 182:45–52. doi: 10.1016/j.theriogenology.2022.01.030, PMID: 35123310

