# Supplementary material for: Variations in bacterial profiles associated with semen collection timing and bull breed, analyzed using 16S rRNA sequencing and MALDI-TOF MS
Source: Front Vet Sci. 2025 Sep 5;12:1583136. doi: 10.3389/fvets.2025.1583136 (PMC12447730; doi:10.3389/fvets.2025.1583136)
Supplement: Supplementary file 1 [file Table_1.docx]

**Supplementary Table 1.** Primer combination and thermal cycling conditions used amplify V3-V4 regions of 16S rRNA.

| **Illumina Primer 16S rRNA** | **Sequences (5’-3’)** | **Terminal Cycling** |
| --- | --- | --- |
| 341F | ACACTCTTTCCCTACACGACGCTCTTCCGATCTCCTACGGGNGGCWGCAG | (98 °C 3 min); (98 °C 30 s, 55 °C 30 s, 72 °C 40 s) × 25; 72 °C 10 min; 10 °C hold |
| 805R | GTGACTGGAGTTCAGACGTGTGCTCTTCCGATCTGACTACHVGGGTATCTAATCC | (98 °C 3 min); (98 °C 30 s, 55 °C 30 s, 72 °C 45 s) × 8; 72 °C 5 min; 10 °C hold |
