# Supplementary material for: Variations in bacterial profiles associated with semen collection timing and bull breed, analyzed using 16S rRNA sequencing and MALDI-TOF MS
Source: Front Vet Sci. 2025 Sep 5;12:1583136. doi: 10.3389/fvets.2025.1583136 (PMC12447730; doi:10.3389/fvets.2025.1583136)
Supplement: Supplementary file 3 [file Data_Sheet_1.docx]

Supplementary Material


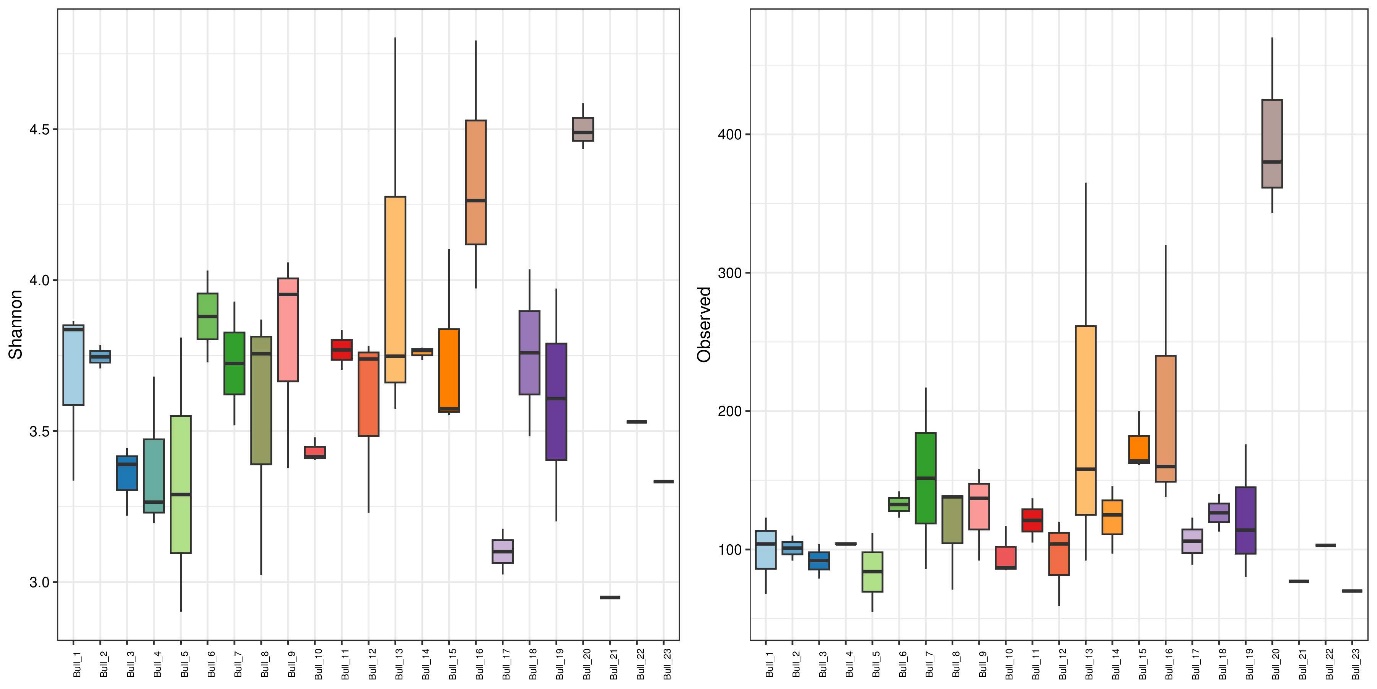


**Supplementary Figure 1.** Alpha diversity measures of semen samples. (Left) Distribution of Shannon index values between the bulls; (Right) Distribution of observed amplicon sequence variants (ASVs) between the bull samples. The horizontal line in the box indicates the median.


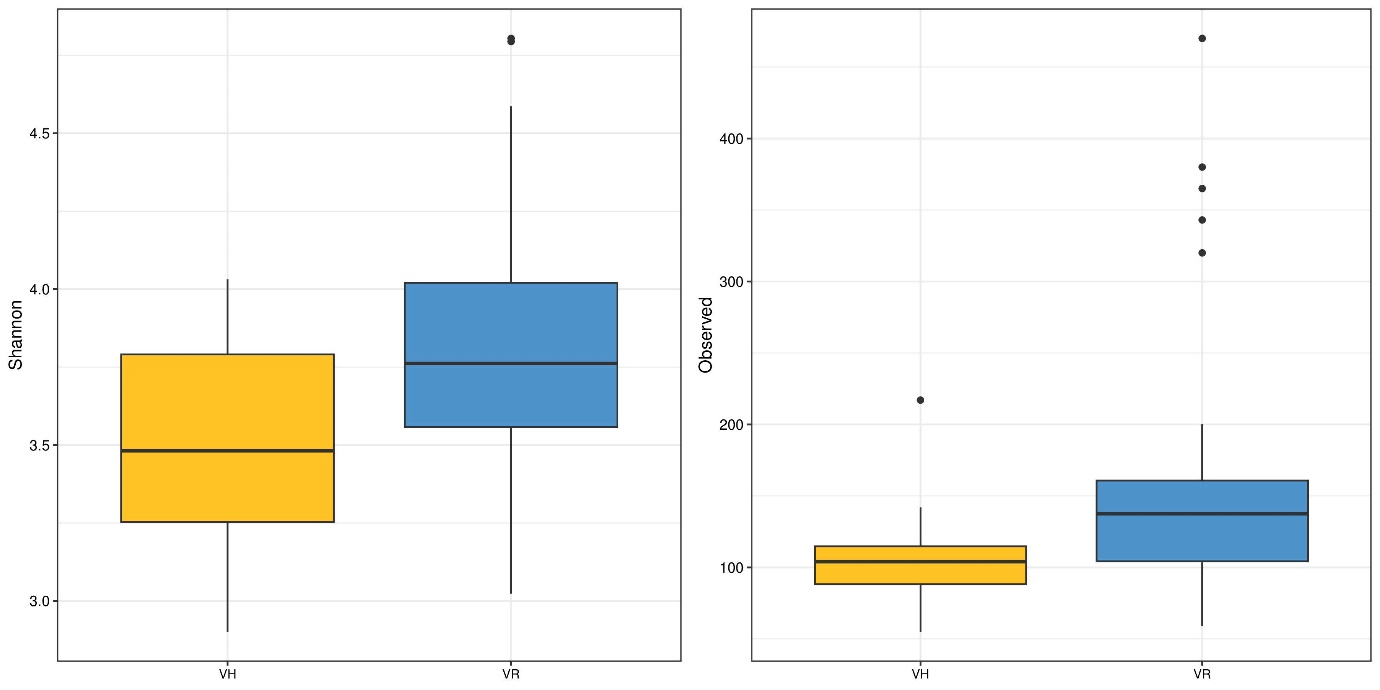


**Supplementary Figure 2.** Alpha diversity measures of samples by grouping them based on their breeds (VH and VR). (Left) Distribution of Shannon index values between the VH and VR breeds; (Right) Distribution of observed amplicon sequence variants (ASVs) between the two breeds. The horizontal line in the box indicates the median.


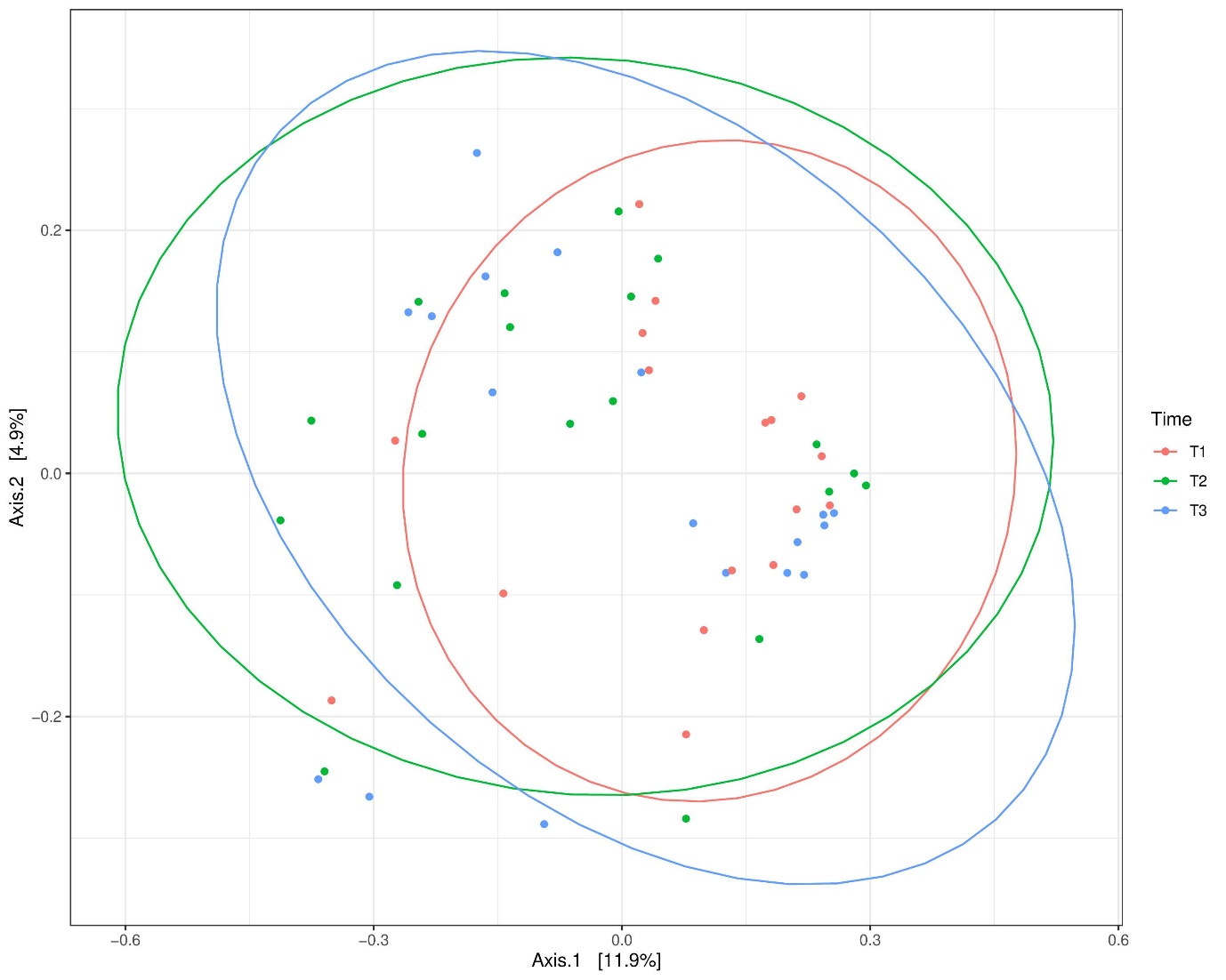


**Supplementary Figure 3.** PCoA plot depicting Bray-Curtis pairwise distances between samples. Axes indicate the proportion of variability explained by that axis. Each colored dot represents the collection time of the sample for each bull.


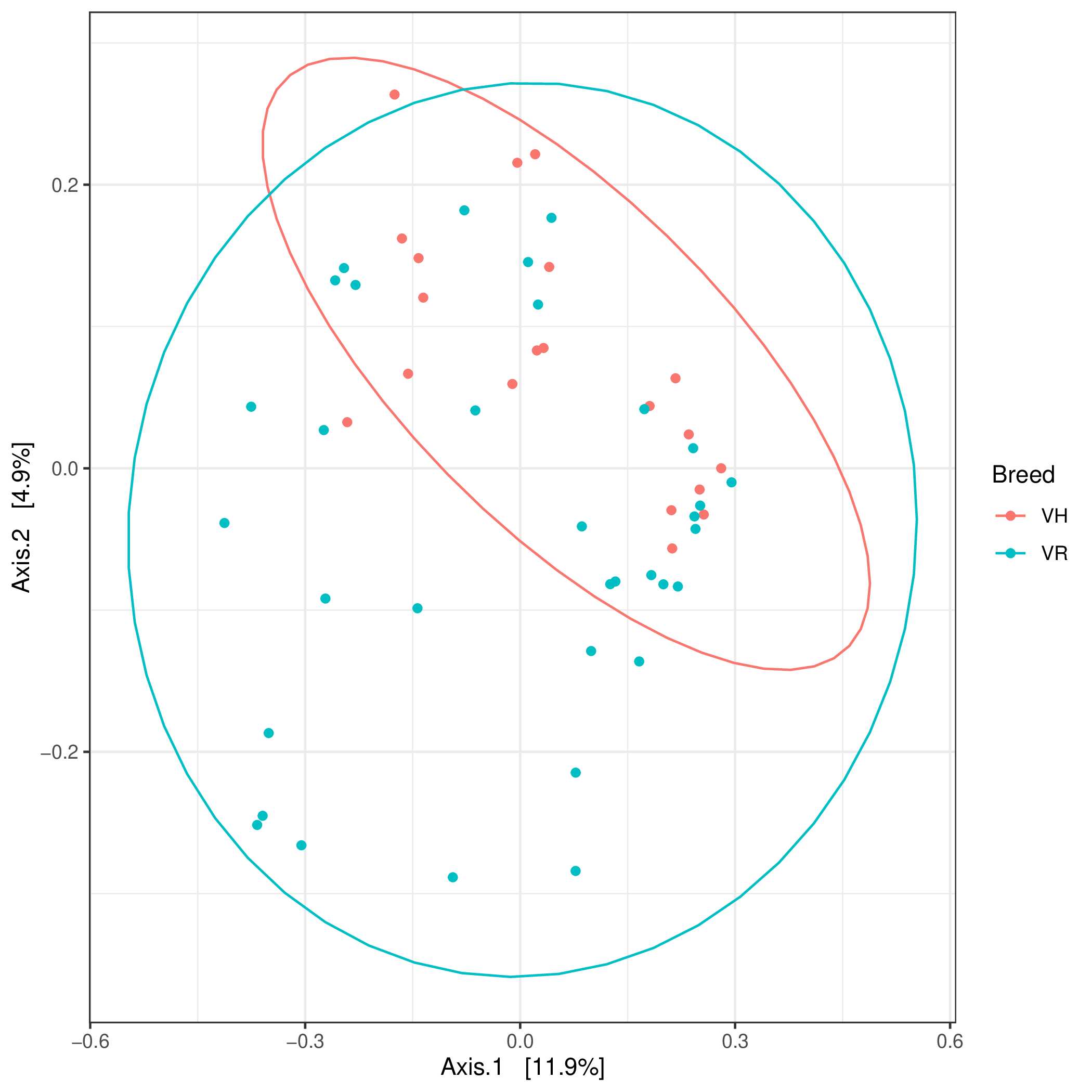


**Supplementary Figure 4.** PCoA plot showing Bray-Curtis pairwise distances between the bull samples categorized by their breeds, VH (red) and VR (blue).
